# Food Safety Practices and Stunting among School-Age Children—An Observational Study Finding from an Urban Slum of Bangladesh

**DOI:** 10.3390/ijerph19138044

**Published:** 2022-06-30

**Authors:** Kazi Istiaque Sanin, Ahshanul Haque, Baitun Nahar, Mustafa Mahfuz, Mansura Khanam, Tahmeed Ahmed

**Affiliations:** 1 Nutrition and Clinical Services Division, icddr,b, Dhaka 1212, Bangladesh; ahshanul.haque@icddrb.org (A.H.); baitun@icddrb.org (B.N.); mustafa@icddrb.org (M.M.); mansura@icddrb.org (M.K.); tahmeed@icddrb.org (T.A.); 2 James P. Grant School of Public Health, BRAC University, Dhaka 1212, Bangladesh; 3 Office of the Executive Director, icddr,b, Dhaka 1212, Bangladesh

**Keywords:** food safety, stunting, school-age children, Bangladeshi slum

## Abstract

**Background:** Food safety incorporates the handling, preparation, and storage of food materials in ways that prevent foodborne illness. We aimed to investigate the typical food safety practices in a Bangladeshi slum context and to explore if stunting among school-age children was associated with various components of food safety. **Method:** We analysed the MAL-ED birth cohort data from the Bangladesh site. A total of 265 healthy children were enrolled in the study; we could follow up and collect food safety-related data from 187 participants. **Results:** The average age of the children was 6.5 years (standard deviation or SD 0.04) and 49% of them were female. About 26% of the children were stunted. In our bivariate analysis, caregivers’ handwashing practice after using the toilet, treatment of drinking water, presence of insects/pests in the cooking area, and child’s eating ready-made/street food more than three times per day were significantly associated with stunting. After adjusting for pertinent factors, treatment of drinking water (adjusted odds ratio or AOR = 2.50, 95% confidence interval or CI: 1.03, 6.05), and child’s eating ready-made/street food more than three times/day (AOR = 2.34, 95%CI: 1.06, 5.15) remained significantly associated with stunting. **Conclusions:** Diverse aspects of food safety practices have a substantial association with stunting among school-age children living in an unhygienic slum environment in Dhaka, Bangladesh.

## 1. Introduction

Stunting, the most prevailing form of child undernutrition, is defined as a height-for-age Z-score of more than 2 standard deviations below the World Health Organization (WHO) Child Growth Standards median [1], indicating not only a restriction of a child’s potential growth and well-being [2], but also inequalities in human development [3]. It affected an estimated 149.2 million children under five years of age in 2020 [4]. This is the marker for chronic deprivation in early childhood, causing increased mortality, impaired cognitive development, and reduced adult income [3]. Several risk factors for stunting have been identified (e.g., foetal growth restriction, short maternal height, inadequate maternal education, infections, and micronutrient deficiencies) [5,6], but causal pathways are yet to be clearly defined [7]. However, quality of food and hygiene practices might play a role in malnutrition among a population living in susceptible environments such as slum areas. Unhygienic preparation and handling of foods is a major source of gastrointestinal diseases, as an immature immune system renders young children vulnerable to foodborne pathogens [8,9]. Diarrhoea and other similar diseases are directly linked with malabsorption of macro and micro nutrients, fluid losses, and reduced appetite [10], consequently resulting in several childhood nutritional problems, such as wasting and stunting [11].

Food safety and hygiene is concerned with the handling, preparation, and storage of food materials in ways that prevent foodborne illness [12]. More than 200 known diseases are transmitted through food alone [13]. The 2015 WHO report [14] on the estimates of the global burden of foodborne diseases reported approximately 600 million foodborne illnesses and 420,000 deaths in 2010. Even more frightening, 40% of the foodborne disease burden was among children under five years of age [14]. The highest burden per population was observed in Africa, followed by South East Asia, continents with the highest burden of stunting among under-five children [4]. Poor sanitation can have a negative impact on young children’s nutritional condition, not only due to decreased nutrient absorption, but also due to subclinical illnesses with faecal pathogens [15]. Repeated diarrheal episodes, soil-transmitted illnesses (helminths), and environmental enteropathy have all been linked to poor WASH and stunting [16].

Environmental enteropathy or enteric dysfunction (EED) is an abnormal subclinical condition of the gut found among the population that usually lacks access to safe water, improved sanitation, and proper hygiene [17,18]. The prolonged and persistent presence of pathogens in the environment and subsequent ingestion through contaminated food and water induces chronic intestinal inflammation, loss of villous surface area, and impaired barrier function resulting in impaired food and nutrient uptake [19]. Researchers have hypothesized that such abnormality of gut function might be the perpetrator behind unsatisfactory results of nutrition interventions to normalise early childhood growth [18]. Improvements to drinking water quality, sanitation, and environmental and personal hygiene have been posited to improve the effectiveness of nutrition interventions by reducing gut dysfunction and thus aid to tackle a substantial portion of the observed growth deficit [20].

However, in Bangladesh, there is still a long way to go to ensure clean water and environment, mostly for the poorest of the poor, who are also most vulnerable to chronic malnutrition. The health status of slum-dwelling people is far worse than those who live in the villages. This population explicitly suffer as they live in overcrowded areas, lacking access to nutritious food, clean drinking water, and proper sanitation. Taking into account the greater risk, we aimed to investigate the typical food safety practices in a Bangladeshi slum context regarding “If certain food safety practices are associated with the stunting among school-age children (>6 y), living in a Bangladeshi slum”.

## 2. Materials and Methods

### 2.1. Study Setting and Population

The Malnutrition and Enteric Disease (MAL-ED) study is a multi-country research activity [21] in which Bangladesh is one of the field sites among the eight countries. For this analysis, we used the MAL-ED birth cohort data from the Bangladesh site [22]. The study was conducted in the Bauniabadh area of Mirpur, an impoverished urban settlement in Dhaka, Bangladesh. We enrolled and followed a total of 265 healthy children born in the catchment area starting in February 2010. Among these 265 children, we could follow-up and collect food safety-related data from 187 participants, based on their presence at home. The rest of the participants were not traceable as they moved out from this slum residence to another place over the years. Caregivers who had no plans to move out of the catchment area for at least 6 months after enrolment and who were willing to be visited at home on a monthly basis were the inclusion criteria of this study. Maternal age of 16 years, not a singleton pregnancy, another child already enrolled in the MAL-ED study, severe illness of the child (who is supposed to be enrolled) requiring hospitalization prior to recruitment, and severe acute or chronic conditions of the child diagnosed by a physician (e.g., neonatal disease, renal disease, chronic heart failure, liver disease, cystic fibrosis, and congenital conditions) were all exclusion criteria for cohort recruitment. All children born in the study community who met all of the inclusion criteria and none of the exclusion criteria were invited to participate in the study.

### 2.2. Data Collection

Field research assistants collected socioeconomic and household-related information including each child’s date of birth, sex, and birth weight (if available) at enrolment using a structured questionnaire. Data including anthropometry and morbidity were collected monthly from the first month and dietary information was collected monthly starting from 9 months onwards, as all the children had transitioned complementary feeding by then (supplementary file). Children were weighed with minimum clothing using a digital scale with 10 g precision (Seca, model no.345, Hamburg, Germany) and height was measured using a measuring board (Seca Infantometer, model no.417, Hamburg, Germany) to the nearest 1 mm according to standard anthropometric methodology [1]. Weighing and length measuring equipment were calibrated daily with standard weights and measured rods to maintain measurement quality, and refresher training was offered on a regular basis. Food safety-related information was collected only once based on a standard structured questionnaire at the end of the follow-up of the cohort. Food safety practices were reported mainly by the mothers. For a very few numbers of participants, caregivers such as grandmothers reported the practice where the mother was working outside the household. We collected dietary data using standard 24 recall food questionnaire. The 24 h recall interview was conducted by research personnel who were educated by expert dietitians and used visual aids (standardized household measurement tools and food photos of varied portion sizes) to help the mothers assess their child’s nutritional intake. To avoid bias, the interviews were held on non-consecutive days and the participants were not informed in advance [23].

### 2.3. Operational Definitions

Improved toilet: This includes a flush toilet, connected to a piped sewer system, connected to a septic system, flush/pour-flush to a pit latrine, or a pit latrine with a slab.

Minimum dietary diversity: Eating at least 4 or more food groups out of 7 food groups in the previous 24 h period [24].

School-age children: this represents children who are eligible to attend a school, usually around the age of six years and above.

### 2.4. Variable Selection for Analysis

We selected explanatory variables based on determinants of stunting reported in the previous literature from both Bangladesh and other low- and middle-income countries (LMICs) [6] and the availability of data from our study. Maternal education has been categorized into 3 groups as no schooling, minimum 5 years, and more than 5 years of schooling. Toilet with flush facility and pit latrine with slab have been coded as improved toilet. Dietary data has been presented into MDD score, which was categorized into 2 groups. Food safety variables were collected using standard questionnaires with five key variables. The core information of the Five Keys to Safer Food were: (1) keep clean; (2) separate raw and cooked; (3) cook thoroughly; (4) keep food at safe temperatures; and (5) use safe water and raw materials. The variables of interest in our study are provided in **Table 1**.

### 2.5. Ethical Statement

The study protocol was reviewed and approved by the Ethical Review Committee of the icddr,b (International Centre for Diarrhoeal Diseases Research, Bangladesh). Informed written consent was obtained from the parents or legal guardians of the participants enrolled in the study.

### 2.6. Statistical Analysis

We used Stata software release 13 (StataCorp, College Station, TX, USA to analyse the data upon entry. The mean and standard deviation are reported for continuous variables and frequency distribution for categorical variables. We compared differences in the mean of continuous variables with normal distribution between groups using Student’s *t*-test after verifying the equality of variance (Levene’s test). The differences in proportion were compared using a Chi-square test or the Fisher’s exact test if the expected number in any expected frequency was <5. Multicollinearity between independent variables was investigated using the variance inflation factor (VIF) values. We constructed separate models using known variables associated with stunting and food safety variables where stunting at around 6 years of age was the outcome variable. Then, we combined those models to investigate if the effect of food safety variables persists even after controlling for the known risk factors. We selected the best model based on the lowest Akaike information criterion (AIC) and Bayesian information criterion (BIC) values.

## 3. Results

### 3.1. Background Characteristics

We could collect data from 187 children out of 265 children originally enrolled in the cohort part of the MAL-ED study; the rest of them were lost to follow-up. During their last follow-up, when data for this analysis was collected, the average age of the children was 6.5 years (SD 0.04) and 49% of them were female. Their mean height was 110 cm (SD 0.41) and mean weight was 17.8 kg (SD 0.24). Of them, 26.2% children were stunted, 12.7% were wasted, and 38.5% were underweight. **Table 2** describes the background characteristics of the participants.

### 3.2. Food Safety Practices According to Stunting Status

More than 80% of the mothers (87%) mentioned that they test if meat, fish, or poultry are fully cooked or not. Covering the food was the main preservation method both for cooked food (81%) and leftover food (70%) and only 22% of the mothers refrigerated the leftover food for the next serving. One-third of the mothers (30%) cooked in definite kitchen space and 27% of the mothers did their cooking near open sewage. The majority of the mothers (78%) washed their hands after using the toilet, and 70% of mothers washed their hands after cleaning the child after defecation. Exclusively all of the respondents used supply water for cooking/drinking purposes. Among them, 77% treated the water by boiling or filtering it before drinking. Almost all the mothers (97%) reported that their children ate ready-to-eat food outside the home regularly. Additional information regarding various food safety-related behaviour is given in **Table 3**.

### 3.3. Association between Food Safety Behaviour and Stunting

In our bivariate analysis, we found that the mother/caregiver’s handwashing practice after using the toilet, treatment of drinking water, presence of insects/pests in the cooking area, and child’s eating ready-made/street food more than 3 times per day, were factors significantly associated with stunting among school-age children. Children whose mother/caregiver(s) never or sometimes washed their hands after using the toilet had more than 2 times greater odds of being stunted (OR = 2.52, 95%CI: 1.21, 5.27) compared to those children whose mother/caregiver(s) always washed their hands. Children from the households who did not treat their drinking water had 2.6 times greater odds of being stunted compared to those who treated the water before drinking (OR = 2.62, 95%, CI: 1.27, 5.43). However, in the multivariable analysis after adjusting for other factors, treatment of drinking water (AOR = 2.50, 95%, CI: 1.03, 6.05), and child’s eating ready-made/street food more than 3 times per day (AOR = 2.34, 95%, CI: 1.06, 5.15) remained significant risk factors for stunting. Among the other two factors, the presence of insects/pests in the cooking area (AOR = 2.16, 95%, CI: 0.98, 4.78) was marginally significant with a *p*-value of 0.057, whereas the mother/caregiver’s handwashing practice after using the toilet was statistically insignificant (AOR = 1.50, 95%, CI: 0.57, 3.92) with a *p*-value of 0.413 in the multivariable analysis. The final multivariable regression model is presented in **Table 4**.

## 4. Discussion

Food safety is a major concern in the usual context of Bangladesh, and the slum environment in the country is far worse. We aimed to investigate the typical food safety practices in a Bangladeshi slum context and to explore if stunting among school-age children residing in the slum area was linked to various components of food safety measures. In our analysis, we found that treatment of drinking water and a child’s eating behaviour outside the home were significantly associated with stunting among school-age children living in a slum area. Furthermore, though statistically marginally significant, the presence of insects/pests in the cooking area was also associated with stunting in the same group of children with moderate effect size.

Bangladesh is among one of the most densely populated countries in the world with unprecedented rapid growth in the urban slum population. Approximately 2.23 million people live in slums across the country with 1.06 million people living in slums in the Dhaka division alone [26]. As per this UN report, children living in slums of Bangladesh are the most disadvantaged and worst-performing in terms of women and children’s health and wellbeing compared to both rural and non-slum urban areas. Bangladesh national micronutrient survey 2013 [27] reports that almost half of preschool-age children living in the slums are stunted compared to urban areas (51.1% vs. 31.3%). Children in urban slums not only come from more food-insecure families but also live in a threatening environment which increases the risk of infection and disease manifolds, making them susceptible to undernutrition.

The use of safe water for drinking and preparing food is a key component of food safety. The impact of clean water, sanitation, and hygiene (WASH) on stunting as separate interventions or in combination has been extensively investigated globally with varied results [28,29]. We found that drinking untreated water was a major risk factor associated with stunting among our study population. In a recent study, researchers collected water samples from four slum areas in Dhaka and found that ninety-nine percent of water used by the slum population contains faecal contamination [30,31]. They tested the water samples from the slums having supply water as the main source of water, similar to our study area. In a previous Bangladeshi study, children younger than 4 years of age living in households with good water quality, improved toilets, and handwashing facilities had a greater height-for-age Z-score (HAZ) score compared to those who were not privileged. In Indonesia, a country burdened with a high prevalence of stunting similar to Bangladesh, the researchers found untreated drinking water combined with unimproved latrines to be associated with an increased odds of stunting among children under 2 years [32]. Other studies have also indicated improved growth outcomes in children from households with either improved water supply, sanitation, or both in several countries [33,34,35]. A systematic review [29] on the impact of WASH interventions on growth in children from low–middle income countries reported an association between improved water supply and quality with a slightly higher weight-for-age Z-score without any evidence of impact on other anthropometric measures or non-diarrheal morbidity. On the other hand, Luby et al. [20] carried out one of the largest cluster-randomized trials in rural areas of Bangladesh to investigate the WASH benefits. They found no effect on the linear growth of children whose households received interventions in terms of chlorinated drinking water, sanitation improvements, or improved handwash practice alone or in combination compared to the control group. They concluded that either the assumption that exposure to faecal contamination contributes significantly to stunting in Bangladesh is flawed, or the delivered interventions in their trial were not able to reduce exposure to environmental pathogens amply to reduce growth faltering. They further suggested that the result might differ in settings with a different prevalence of gastrointestinal disease [36]. Inconsistent findings of the effect of water, sanitation, and hygiene might be explained by discrepancies in hygienic conditions of the environment of the child as well as personal hygiene practices.

We found that children who ate ready-made food (also known as street food) more frequently had higher odds of stunting. By definition, “street food” describes a wide range of ready-to-eat foods and beverages sold and sometimes prepared in public places, notably streets [37]. Like many other countries, street food is the least expensive and most accessible means of the meal outside the home, particularly for low-income people in Bangladesh [38]. Street food plays an imperative socio-economic role in meeting food and nutritional requirements of city consumers at affordable prices [39]. However, due to questionable knowledge and perception regarding safe food handling, unsanitary environmental conditions (such as proximity to sewers and garbage dumps), lack of safe water, sanitation, and hygiene, street food is perceived as a public health risk [39]. Street food in Bangladesh is typically prepared and processed manually and sold in open spaces, by the roadside or by itinerant vendors [38]. A study by FAO on street food vendors in Dhaka city demonstrated that nearly 70% of the vending shops were located on the footpath and one-third of the vending carts were situated near the municipal drain and 18% near the sewerage. They further reported a high prevalence of aerobic bacteria, coliform bacteria, and pathogens in different food items, drinking water, and hand swab samples [40]. Data on street food and its consumers are scarce, but children and school-going students are supposedly major consumers of such foods [38]. Although all the mothers in our study perceived eating such street food to be associated with illness, routine intake of street food suggests that such behaviour is presumably going to persist anyway. Studies suggest that consumers who are attracted by the convenience and low prices of street food are more likely to overlook aspects of hygiene or sanitation, mostly the people from low socio-economic groups [39,41].

In our study population, unimproved toilet type and the presence of insects near the cooking area were found to be associated with stunting among school-age children. Although not statistically significant, the moderate strength of the effect sizes in our analysis indicates their significance as a part of the environmental factor. The pathways linking food safety to stunting are complex, encompassing multiple direct biological routes and several other less direct routes. Among them, the role of the environment on undernutrition has garnered much attention and subsequent evidence in recent years. Previously, the role of diarrhoea on linear growth has been studied extensively [42]. However, in recent years it has been proposed that EED may be much more common than explicit diarrheal illness [43], especially among those living in unhygienic environments [44]. Therefore, researchers now argue that EED, and not diarrhoea, maybe the primary causal mechanism linking environment and hygiene to child growth which might further explain the sub-optimal effectiveness of nutritional interventions to improve growth in developing countries [18,45,46]. Even when children are not apparently infected, the microbial-laden environment may provide a low-level chronic immune stimulation with catabolic consequences that alters the gut in a way that adversely affects the health status [17]. Lin et al. carried out their study in a rural area of Bangladesh where they found that children living in households with an unhygienic environment are both more likely to have EED, and are more likely to be stunted [46]. A study based on the MAL-ED Bangladeshi birth cohort to which our study population belongs has reported the presence of a high level of Myeloperoxidase (MPO), a faecal biomarker of EED among the children which was positively associated with reduced linear growth in their second year of life [47]. From the same study population, Fahim et al. further reported a significant association between faecal MPO and giardiasis, the most common protozoan to cause intestinal parasitic infection during the first 2 years of life ensuing growth faltering [48]. Therefore, it has been suggested to formulate and implement policies to improve perception and practice related to food safety, including personal and environmental hygiene, to reduce environmental enteropathy, a potential cause of stunting in Bangladesh [49].

### Strengths and Weaknesses of the Study

Although our study participants came from an established birth cohort, the information related to food safety was collected only once in a cross-sectional manner. Hence, we could not establish causality from our study findings. However, as we have collected information on all the children since birth, we were able to adjust for most of the well-known factors associated with stunting. Therefore, we infer that the relationship that we found between food safety practices and stunting among school-age children is valid considering the context.

The food safety practices reported in this study were collected only once and based on self-reporting rather than direct observation. It is well-established that people commonly over-report socially desirable behaviour and under-report stigmatized behaviour [50]. Even if behaviour such as handwashing practice and treatment of drinking water, which are socially desirable, were over-reported, we found them to be risk factors for stunting in our bivariate or multivariable analyses, suggesting that the true picture might be far worse and the association might be even sturdier in absence of any reporting bias. Another limitation of the study is the data on food safety practices were collected just once. Therefore, no precise conclusions can be drawn about the safety of the food that the children ultimately consumed.

## 5. Conclusions

Our findings indicate that different aspects of food safety practices have a significant association with stunting among school-age children living in an impoverished and unhygienic slum environment in Dhaka, Bangladesh. Even though the link between an unhygienic environment and growth faltering is being studied with added inquisitiveness in recent years, other aspects of food safety practices should also be investigated robustly considering the usual grimy slum context. Researchers and policymakers might have to come up with different innovative plans explicitly designed for slum areas to mitigate the burden of stunting among the vulnerable population and as a whole in the country.

## Figures and Tables

**Table 1:** Variables of interest.

Known Risk Factors	Food Safety Variables
**Child factors** Age, sex, birth weight, duration of exclusive breast feeding, minimum dietary diversity, minimum meal frequency, minimum adequate diet, and morbidity **Maternal factors** Maternal age, maternal height/weight, and maternal education level **Social factors** Drinking water source, presence of improved toilet, and household asset	Five keys to safer foods based on WHO recommendation [25] Keep clean; Separate raw and cooked; Cook thoroughly; Keep food at a safe temperature; Use safe water and raw materials.
**Outcome variable:** Stunting among school-age children	

**Table 2:** Background characteristics of the study population (n = 187).

Indicators	Stunted (n = 49)	Not-Stunted (n = 138)
**Average age of the children (year)**	6.4 ± 0.08	6.5 ± 0.05
**Female: male ratio**	1:0.9	1:1.04
**History of low birth weight (%)**	44.9	23.2
**Average age of the mothers (year)**	24.7 ± 0.86	25.1 ± 0.39
**Mother’s education level (%)**		
No schooling	28.6	15.3
Less than 5 years	36.7	44.9
More than 5 years	34.7	39.8
**Average monthly income (USD)**	112.5 ± 16.6	118.2 ± 6.3
**No of person per room**	4	3
**Improved toilet (%)**	75.5	90.6
**Mean illness duration in past 30 days (days)**	5.3 ± 0.5	5.1 ± 0.3
**Mean Height (cm)**	105.12 ± 0.48	113.04 ± 0.40
**Mean Weight (kg)**	15.21 ± 0.18	18.74 ± 0.29

**Table 3:** Food safety practices **^a^** according to stunting status.

	Not-Stunted (n = 138)	Stunted (n = 49)	*p*-Value ^b^
**Keep clean**			
**Mother/caregiver washes hand**			
Before handling food materials	113 (81.9)	38 (77.6)	0.509
Before preparing meal	107 (77.5)	33 (68.8)	0.224
Before eating	135 (97.8)	49 (100)	0.568
After handling raw meat/fish	132 (95.6)	44 (89.8)	0.134
After using the toilet	114 (82.6)	32 (65.3)	0.012
After cleaning child after defecation	100 (72.5)	31 (63.3)	0.227
Using soap and water	113 (81.9)	47 (95.9)	0.017
**Mother/caregiver cleans utensils**			
before using them	134 (97.1)	49 (100)	0.228
after using with raw meat, fish, or poultry	138 (100)	49 (100)	
using soap/power	138 (100)	49 (100)	
Mother/caregiver rewashes hands after interruption	61 (44.2)	27 (55.1)	0.189
Cook in a kitchen	47 (34.1)	9 (18.4)	0.039
Cook near sewage	32 (23.2)	19 (38.8)	0.035
Insects/pets always present near cooking area	75 (54.3)	36 (73.5)	0.019
**Separate raw and cooked**			
Stores raw materials before cooking	117 (84.8)	38 (77.6)	0.248
Store food in a container	131 (94.9)	48 (97.9)	0.683
Store cooked food using cover	113 (81.9)	37 (77.1)	0.688
**Cook thoroughly**			
Tests if fully cooked (Yes)	125 (90.6)	37 (75.5)	0.008
Method of testing cooked food (spoon)	113 (92.6)	33 (84.6)	0.201
**Keep foods at safe temperature**			
keep cooked food at room temperature	136 (98.5)	47 (95.9)	0.688
Have leftover food after eating	134 (97.1)	47 (95.9)	0.653
Preserve leftover food for later	134 (97.1)	47 (95.9)	0.653
Refrigerate leftover food	30 (21.7)	10 (20.4)	0.342
Refrigerate fruits/vegetable	95 (68.8)	25 (51.0)	0.047
**Use safe water and raw materials**			
Offer child raw fruits/vegetable	136 (98.6)	48 (98.0)	1.000
Wash fruits/veg before eating	137 (99.2)	49 (100)	1.000
Water for cooking/drinking (supply)	138	49	
Treat drinking water (yes)	113 (81.9)	31 (63.3)	0.008
Child eats food outside home (ready-to-eat food) > 3 times/day	73 (54.5)	34 (73.9)	0.021

^a^ Practices are self-reported, not observed; ^b^ Statistically significant differences obtained using Chi-square test.

**Table 4:** Association between food safety behaviour and stunting among school-age children (n = 180).

Factors	OR ^a^, 95%CI	*p*-Value	AOR ^b^, 95%CI	*p*-Value
**Length at birth**	0.73 (0.62, 0.86)	0.000	0.76 (0.62, 0.94)	0.011
**Mother’s BMI**				
≥18.5	Ref		Ref	
<18.5	4.2 (1.78, 9.92)	0.001	3.08 (1.12, 8.50)	0.030
**Toilet type**				
Improved	Ref		Ref	
Unimproved	3.11 (1.31, 7.43)	0.010	2.61 (0.94, 7.25)	0.066
**MDD at 24 mo**				
≥4 groups	Ref		Ref	
<4 groups	3.23 (1.48, 7.03)	0.003	2.62 (1.08, 6.34)	0.034
**Treatment of drinking water**				
Yes	Ref		Ref	
No	2.62 (1.27, 5.43)	0.009	2.50 (1.03, 6.05)	0.043
**Presence of insects/pest in cooking area**				
Never	Ref		Ref	
Sometimes/always	2.33 (1.13, 4.77)	0.021	2.16 (0.98, 4.78)	0.057
**Child eats food outside home**				
≤3 times/day	Ref		Ref	
>3 times/day	2.37 (1.13, 4.98)	0.023	2.34 (1.06, 5.15)	0.036

**^a^** OR—Odds ratio; ^b^ AOR—Adjusted odds ratio.

## Data Availability

Due to restrictions in icddr,b’s data access policy in regard to participants identifying information, data are available upon request from the Research & Clinical Administration and Strategy (RCAS) of icddr,b (http://www.icddrb.org/component/content/article/10003-datapolicies/1893-datapolicies (accessed on 17 May 2022)) for researchers who meet the criteria for access to confidential data.

## Supplementary Materials

The following supporting information can be downloaded at: https://www.mdpi.com/article/10.3390/ijerph19138044/s1, File S1: Flowchart of data collection.

## Abbreviations

AOR	Adjusted odds ratio
AIC	Akaike information criterion
BIC	Bayesian information criterion
CI	Confidence Interval
EED	Environmental enteric dysfunction
HAZ	height-for-age Z-score
LMICs	Low and Low Middle Income Countries
MAL-ED	Malnutrition and Enteric Disease
MPO	Myeloperoxidase
OR	odds ratio
SD	Standard deviation
WASH	water, sanitation, and hygiene
WHO	World Health Organization
