# Supplementary figures and images for: Food Safety Practices and Stunting among School-Age Children—An Observational Study Finding from an Urban Slum of Bangladesh
Source: Int J Environ Res Public Health. 2022 Jun 30;19(13):8044. doi: 10.3390/ijerph19138044 (PMC9265275; doi:10.3390/ijerph19138044)

Flowchart of data collection-

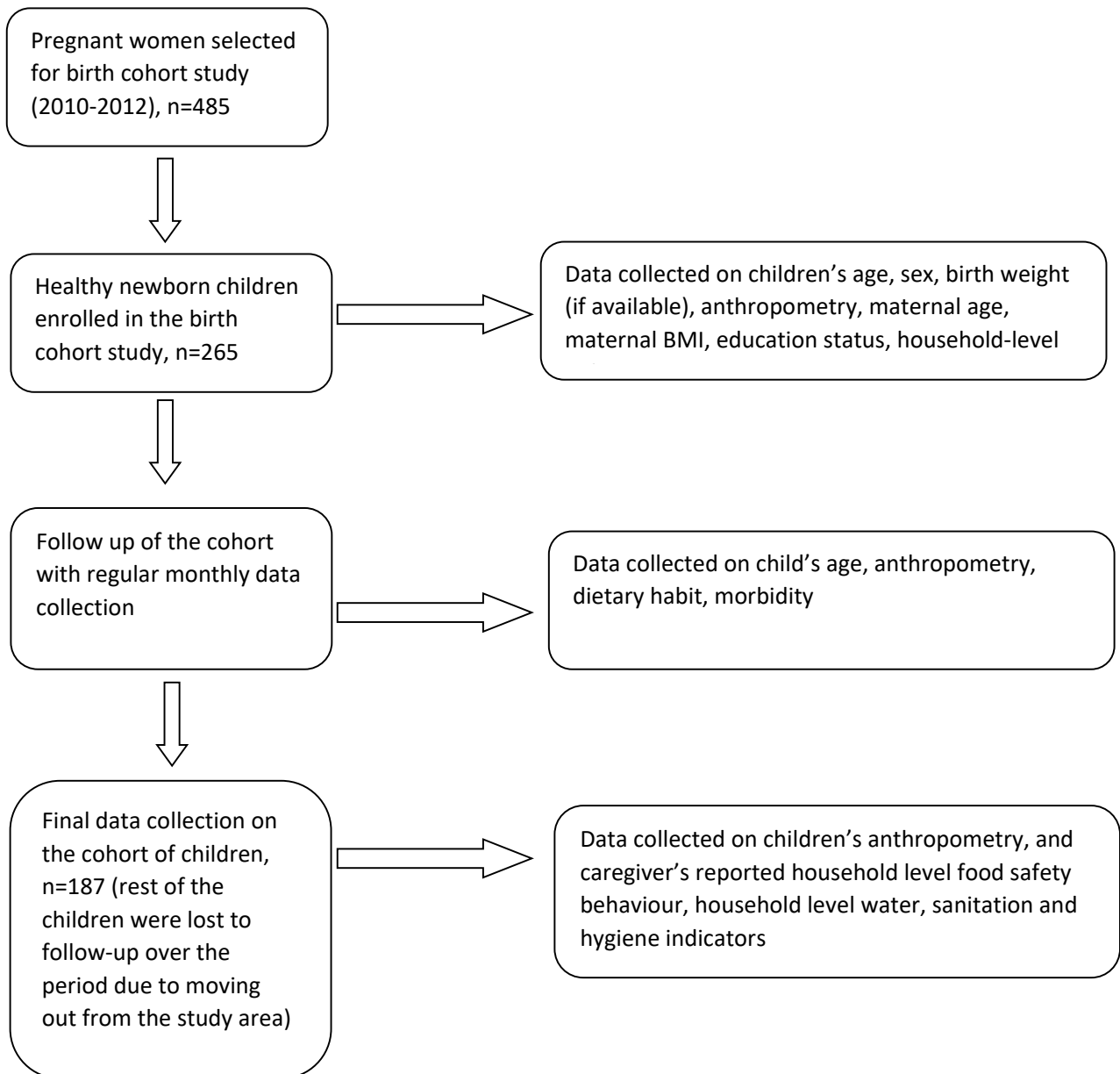

Supplement: Supplementary file 1 [file ijerph-19-08044-s001.zip › ijerph-1753857-Supplementary File S1.pdf]
